# A man with facial disfigurement

**DOI:** 10.11604/pamj.2018.30.196.16299

**Published:** 2018-07-05

**Authors:** Phatharaporn Kiatpanabhikul, Thiti Snabboon

**Affiliations:** 1 Department of Medicine, Charoenkrungpracharak Hospital, Medical Service, Bangkok Metropolitan Administration, Bangkok, Thailand; 2 Excellence Center in Diabetes, Hormones and Metabolism, King Chulalongkorn Memorial Hospital, Bangkok, Thailand; 3 Department of Medicine, Faculty of Medicine, Chulalongkorn University, Bangkok, Thailand

**Keywords:** Fibrous dysplasia, craniofacial, mccune-albright syndrome

## Image in medicine

A 21-year-old man presented with progressive facial disfigurement along with intermittent pain over his prominent mandible and forehead. Examination revealed asymmetrical facial figures especially the forehead and his left side (A). CT skull demonstrated typical features of fibrous dysplasia (FD) including multiple expansile lesions with heterogeneous pattern including radiolucency interspersed with ground glass opacity involving all craniofacial bones (B). No periosteal reaction or soft tissue involvement is noted. Extensive bone survey and hormonal assessments were unremarkable. The patient preferred conservative treatment with pain relief and undergoing regular follow-up. FD is a benign and progressive disorder in which the bone is replaced and expanded by fibro-osseous tissue, leading to fractures and deformities. It may affect a single (monostotic) or multiple (polyostotic) bones. The latter may be associated with endocrine dysfunctions and cafe' au lait spots (McCune-Albright syndrome) or soft tissue myxomas (Mazabraud syndrome). Craniofacial bone involvement has been reported in about one-fourth of the FD case. Its manifestations are varied depending on the involving bones including painless swelling with facial asymmetry, malocclusion, headache, visual or hearing impairment, nasal obstruction or neurological deficit. Diagnosis is based on the clinical ground and typical radiographic findings. Biopsy is reserved for cases suspicious of malignant change or indeterminate diagnosis. Treatment is aimed to alleviate cosmetic or functional problems, ranging from observation, medical treatment with bisphosphonates, to extensive surgery. Regular follow-up is mandatory to detect disease progression or malignant change.

**Figure 1:**
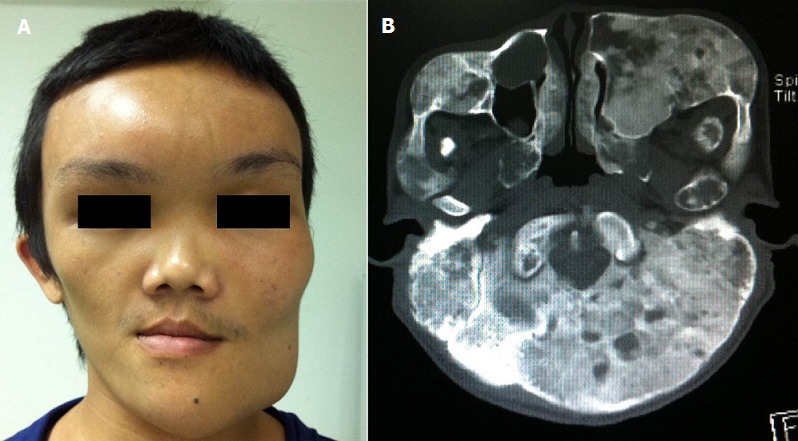
A) the patient picture. Note asymmetrical facial figure especially the forehead and left side; B) a CT scan (axial view). Multiple expansile lesions with a heterogeneous pattern, including radiolucency interspersed with ground-glass opacity of craniofacial bones

